# Stacking Fault Energy Analyses of Additively Manufactured Stainless Steel 316L and CrCoNi Medium Entropy Alloy Using *In Situ* Neutron Diffraction

**DOI:** 10.1038/s41598-020-58273-3

**Published:** 2020-01-28

**Authors:** W. Woo, J. S. Jeong, D.-K. Kim, C. M. Lee, S.-H. Choi, J.-Y. Suh, S. Y. Lee, S. Harjo, T. Kawasaki

**Affiliations:** 1 Neutron Science Center, Korea Atomic Energy Research Institute, Daejeon, 34057 Korea; 2 Materials Technology Development Team, Doosan heavy industries, Changwon, 44610 Korea; 3 School of Mechanical Engineering, University of Ulsan, Ulsan, 44610 Korea; 4 Department of Printed Electronics Engineering, Sunchon National University, Sunchon, 57922 Korea; 5 High Temperature Energy Materials Research Center, Korea Institute of Science and Technology, Seoul, 02792 Korea; 6 Department of Materials Science and Engineering, Chungnam National University, Daejeon, 34134 Korea; 7 J-PARC Center, Japan Atomic Energy Agency, 2-4 Shirakata, Tokai, Naka, Ibaraki, 319-1195 Japan

**Keywords:** Materials science, Physics

## Abstract

Stacking fault energies (SFE) were determined in additively manufactured (AM) stainless steel (SS 316 L) and equiatomic CrCoNi medium-entropy alloys. AM specimens were fabricated via directed energy deposition and tensile loaded at room temperature. *In situ* neutron diffraction was performed to obtain a number of faulting-embedded diffraction peaks simultaneously from a set of (hkl) grains during deformation. The peak profiles diffracted from imperfect crystal structures were analyzed to correlate stacking fault probabilities and mean-square lattice strains to the SFE. The result shows that averaged SFEs are 32.8 mJ/m^2^ for the AM SS 316 L and 15.1 mJ/m^2^ for the AM CrCoNi alloys. Meanwhile, during deformation, the SFE varies from 46 to 21 mJ/m^2^ (AM SS 316 L) and 24 to 11 mJ/m^2^ (AM CrCoNi) from initial to stabilized stages, respectively. The transient SFEs are attributed to the deformation activity changes from dislocation slip to twinning as straining. The twinning deformation substructure and atomic stacking faults were confirmed by electron backscatter diffraction (EBSD) and transmission electron microscopy (TEM). The significant variance of the SFE suggests the critical twinning stress as 830 ± 25 MPa for the AM SS 316 L and 790 ± 40 MPa for AM CrCoNi, respectively.

## Introduction

Excellent combination of strength, ductility, and toughness has been found in an equiatomic, face-centered-cubic CrCoNiFeMn high-entropy alloys (HEA)^1^. The reason of the exceptional properties at cryogenic temperature has been mainly attributed to the evolution of the nanoscale twinning under plastic deformation, so-called twinning-induced plasticity^2,3^. Compared to the HEA, superior mechanical properties of CrCoNi medium-entropy alloys (MEA) have been recently reported at both room and cryogenic temperature^4–13^. High attention has been focused on the evolution of the twinning substructure and/or a new phase with hexagonal close packed structure instead of the initial deformation mode of the dislocation slip in MEAs^6–9^. Systematic examinations of the substructure elucidate that the critical twinning stress of 790 ± 100 MPa reaches at the earlier strain of 9.7–12.9% for CrCoNi MEA than 720 ± 30 MPa at ~25% for CrCoNiFeMn HEA because of higher yield strength and work hardening rate with larger shear modulus of the MEA^8–10^. Earlier formation of the nano-twinning and its activation over a more extended strain range is of importance accepted as the reason of the exceptional strength-ductility-toughness combination in MEA.

Stacking fault energy (SFE) has been accepted as a responsible parameter to determine the deformation schemes, which is typically by slip (>45 mJ/m^2^) to twinning (20–45 mJ/m^2^) and/or phase transformation (<20 mJ/m^2^) as often reported in austenitic stainless steels^14–21^. The SFE is defined as the energy per fault area by dissociating a perfect dislocation into Shockley partial dislocations and considered as a surface tension pulling the partials, which is inversely proportional to the equilibrium distance between two partials^14^. The key issue of the CrCoNi MEA is the low SFE, which creates a wide stacking fault ribbon limiting the cross slip deformation mode and provides the superior mechanical properties by the dominant deformation twinning^6–11^. Up to date, Laplanche *et al*.^9^ reported the SFE of 22 ± 4 mJ/m^2^ in CrCoNi MEA, which is ~25% lower than CrCoNiFeMn HEA (30 ± 4 mJ/m^2^) and Liu *et al*. reported as 18 ± 4 mJ/m^2^ in CrCoNi MEA and 26.5 ± 4.5 mJ/m^2^ in CrCoNiFeMn HEA using TEM^2,11^. Besides, a number of *ab initio* calculations provide mostly negative SFEs (e.g., −26 mJ/m^2^ for CrCoNi MEA, −7 mJ/m^2^ for CrCoNiFeMn HEA by Huang *et al*.^10^), which have been simulated under ignoring or including the temperature dependency at a given configuration of atoms and/or with the chemical fluctuations in a mesoscale level^22–24^. In recent, Wang *et al*. suggested lower SFE of 13 mJ/m^2^ at 77 K than 32.5 mJ/m^2^ at 293 K in CrCoNiFe HEA by using *in situ* neutron diffraction coupled with peak profile analysis^12^.

Additive manufacturing (AM) has attracted much attention over past ten years in the perspective of an innovative fabrication processing including intrinsic design freedom and short lead times^25^. Heat sources (laser or electron beam) of the AM melt metal particles selectively and build up incrementally layer by layer utilizing powder bed fusion (PBF) or direct energy deposition (DED) processes^26,27^. Inherently, the small melting particles (~a few hundreds μm in diameter) experience rapid solidification with fast cooling rates (about 10^6^ K/s in PBF and 10^2^ K/s in DED)^28^. Such higher cooling rates of AM process can provide significantly different microstructural characteristics such as fine grains, directional grain architectures, and non-equilibrium phases/composition substructures compared to the conventional casting process (~0.1–10 K/s)^26–28^. As a result, several studies have reported higher yield strengths and comparable elongations compared to cast or wrought forms in AM stainless steels (SS)^27–30^. Pham *et al*. reported extraordinary high yield strength of 520 MPa and elongation of ~60% in PBF AM SS 316 L (double of annealed commercial SS 316 L alloys) and highlighted fine subgrains having high dislocation density and strong twinning-induced plasticity^28^. Recently AM reaches HEAs, for example, AM CrCoNiFe (Al, Ti, Mn)^31–33^ and AM refractory HEAs (MoNbTaW, TiZrNbTa)^34,35^. Noticeably, Li *et al*. showed tensile strength over 600 MPa in a high energy laser AM CrCoNiFeMn HEA (not less than cast-wrought CrCoNiFeMn HEA) having a large number of dislocation pile-ups and nanotwins in refined grains^33^. Thus, full-fill knowledge and accurate analyses of the SFE is critical to elucidate the reason of the superior strength properties, which is highly relevant to the dominant deformation mode between dislocation slip and twinning in AM alloys.

To determine SFEs, three methods have been generally utilized. TEM imaging technique (e.g., weak-beam dark-filed, WBDF) directly measures spacings between dissociated partial dislocations^9,11,20,21^. Indirect analyses using x-ray^16,17^ and neutron diffraction^12,19^ rely on peak profile analysis of faulting-embedded diffraction patterns. TEM method has difficulties in statistic assurance due to the investigation of localized regions and proper thin sample preparation without grain structure changes. Besides, TEM images are often taken at early loading stages (a few % strain) to avoid any complication from heavy density of dislocations. X-ray diffraction has a limit on widely discrete results due to the surface reflection where is highly depending on sample conditions. Meanwhile, neutron diffraction can provide volume-averaged bulk characteristics among thousands of grains due to the deep penetration capability through the thickness over a few centimeters. Based on the experimental methodologies the analyzed SFEs of the SS 316 L^16–21^ is relatively prevalent as 12.9–42 mJ/m^2^, whereas it is barely found in the cases of CrCoNi MEA (18–22 mJ/m^2^) and CrCoNiFeMn HEA (26.5–30 mJ/m^2^)^2,9,11^. Furthermore, no SFE analysis has been reported in the AM stainless steels and AM HEA/MEAs in literature to date. As mechanical properties of HEA/MEAs are typically compared to those of the stainless steels^1,3,4^, the SFEs of AM SS 316 L and AM CrCoNi MEA are examined thoroughly in this paper.

The purpose of this paper is to reveal (i) mechanical properties including yield/tensile strengths, elongation, and work hardening rates during tensile loading in AM SS 316 L and AM CrCoNi MEA; (ii) elastic and plastic deformation parameters such as diffraction elastic constant and lattice strain evolution of (hkl) grains in bulk AM specimens using *in situ* neutron diffraction; (iii) variations of stacking fault probability, mean-square lattice strain, and SFE as a function of strain analyzed from a total of 83 faulting-embedded diffraction peak profiles; and finally (iv) deformation substructure and atomic stacking including subgrains, texture, dislocations, twins, and stacking faults examined by EBSD and TEM. Thus, this study correlates diffraction peak profiles to SFEs and elucidates the twinning substructure behind outstanding mechanical properties in AM SS 316L and AM CrCoNi MEA.

## Results

### Mechanical properties

**Figure 1:**
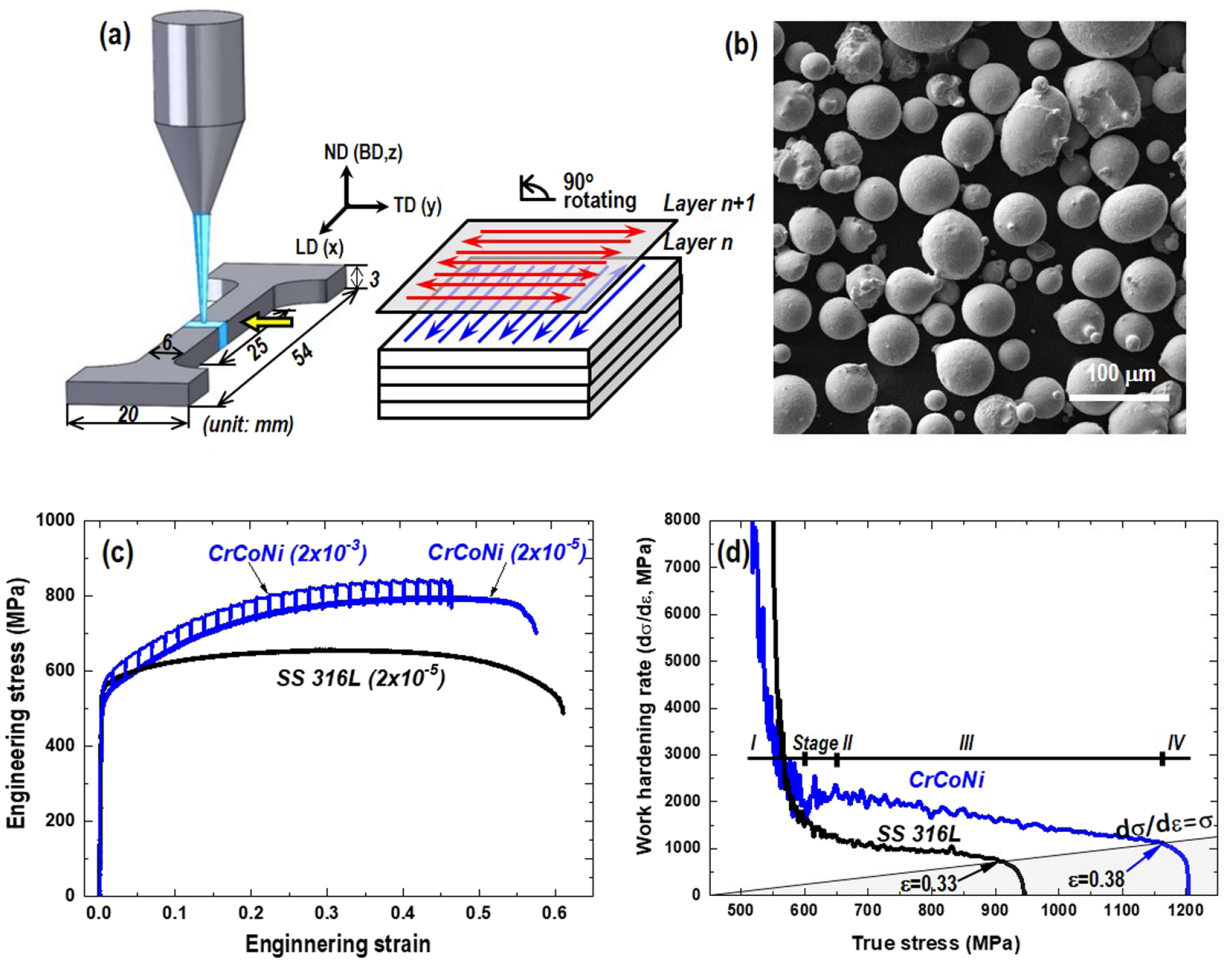
(**a**) Schematic of the sample dimension and orthogonal scanning strategy, (**b**) powder size and morphology for the additive manufacturing (AM) process, (**c**) engineering stress-strain curves of tensile tested with the strain rate of 2 × 10^−5^ s^−1^ for AM stainless steel 316 L and AM CrCoNi specimens. Included tensile test result of 2 × 10^−3^ s^−1^ for AM CrCoNi specimen and (**d**) work hardening rate (d*σ/dε*, MPa) as a function of true stress.

Tensile specimens were additively manufactured by using the DED process using AM powder (see Methods, Fig. 1(a,b)). Figure 1(c) show the engineering stress-strain curve with the strain rate of 2 × 10^−5^ s^−1^ in AM SS 316 L and AM CrCoNi specimens. To avoid complication the results of higher strain rate (2 × 10^−3^ s^−1^) will be described later separately. It shows the yield strength (*σ*_*y*_), ultimate tensile strength (*σ*_*UTS*_), and elongation (*ε*_*f*_) of 540 MPa, 660 MPa, 62% for AM SS 316 L, respectively, as summarized in Table 1. It is higher than typical cast-wrought type SS 316 L specimens (*σ*_*y*_: 260–300 MPa, *σ*_*UTS*_: 500–600 MPa, *ε*_*f*_: 40–50%) and similar to the PBF SS 316 L specimens (550–650 MPa, 580–730, 50–55%) in literature^28,30^. Meanwhile, tensile properties of AM CrCoNi (490 MPa, 790 MPa, 57%) is comparable to those of cast-wrought CrCoNi alloys (360–440 MPa, 800–890 MPa, 46–72%)^4,6,9^. A recent study of the cast-wrought CrCoNi shows wide ranges of *σ*_*y*_ (350–1300 MPa), *σ*_*UTS*_ (800–1300 MPa), and *ε*_*f*_ (15–75%) depending on degrees of recrystallization relevant to twins and dislocation densities^13^. Higher work hardening was observed in AM CrCoNi compared to the AM SS 316 L in true stress-strain curve (shown in Fig. S2, Supplementary information). The hardening capacity (H_c_ = *σ*_*UTS*_*/σ*_*y*_ − 1) of 1.50 in AM CrCoNi is two times higher than 0.75 in AM SS 316 L.

**Table 1:** Mechanical properties of yield strength (*σ*_*y*_, MPa), ultimate tensile strength (*σ*_*UTS*_, MPa), and elongation (*ε*_*f*_, %) in engineering stress-strain curves of the AM SS 316 L and AM CrCoNi alloys.

Material	YS (MPa)	UTS (MPa)	EL (%)	Lattice parameter (initial, nm)	Diffraction elastic constants (GPa)					Possion’s ratio				
					E_bulk_	E_111_	E_200_	E_220_	E_311_	ν_bulk_	ν_111_	ν_200_	ν_220_	ν_311_
AM SS 316 L	540	660	62	0.3596	218	246.3	185.1	213.7	198.3	0.34	0.36	0.43	0.35	0.34
AM CrCoNi	490	790	57	0.3567	235	292.5	170.1	269.2	211.1	0.33	—	0.37	0.38	0.30

Summarized the initial lattice parameter (*a*_*o*_, nm), diffraction elastic constant (*E*_*hkl*_, GPa), and Possion’s ratio (*v*_*hkl*_) of each (*hkl*) plane. The *E*_*bulk*_ and *v*_*bulk*_ were obtained by Rietveld whole peak fitting of the *a*_*o*_ evolutions. Note that the strain rate is 2 × 10^−5^ s^−1^.

Figure 1(d) shows the work hardening rate (WHR, d*σ/dε*) as a function of true stress, which is the derivative of the true stress regarding true strain. The WHR of AM SS 316 L rapidly decreases and remain almost constant around 1000 MPa, whereas AM CrCoNi shows a rapid decrease, rather increases (Stage II), and gradually decreases until fracture. The four-stage response has been known as a characteristic of low SFE fcc metals and the stage II often includes primary twinning and its migration^36,37^ and/or martensitic transformation^38^. Besides, the necking criterion (d*σ/dε* = *σ*) predicts a delayed necking occurrence of AM CrCoNi (38%) compared to the AM SS 316 L (33%).

### Grain structure analyses by EBSD

**Figure 2:**
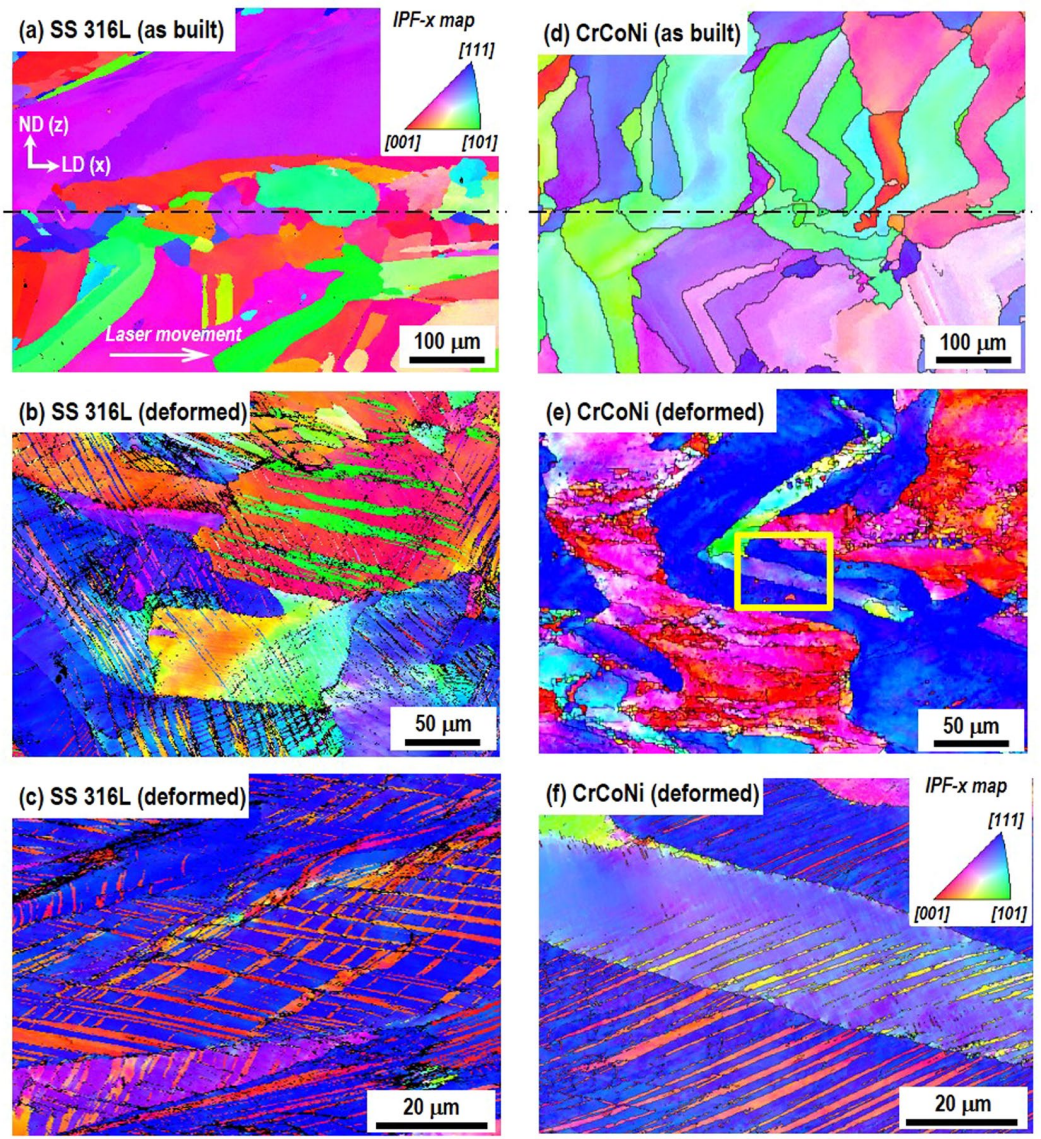
Inverse pole figure (IPF) maps performed at LD-ND plane of the specimen by EBSD and the orientations were analyzed along LD (x) axis (//loading direction, IPF-x map); (**a**) as built and (**b**,**c**) deformed AM SS 316 L, (**d**) as built and (**e**,**f**) deformed AM CrCoNi specimens.

Figure 2 shows inverse pole figure (IPF) maps performed at LD(x)-ND(z) plane marked as an arrow in Fig. 1(a) and represented the crystallographic orientations along LD(x) axis (so called IPF-x map). Firstly, for the as-built AM SS 316 L, Fig. 2(a) shows mostly columnar grain structure grown along the building direction (//ND, z) and the columnar grains are rotated toward LD (x) due to the laser movement during the AM processing. Near the mid-thickness, equiaxed fine grains are observed with the mean grain size of about 33 μm (based on the linear intercept method), which is relatively smaller than the typical grain size observed in cast-wrought SS 316 L (30–60 μm)^26^. It is obtained due to the alternate layer stacking by the orthogonal scanning strategy examined from the perpendicular plane to the building direction^27^. In contrast, Fig. 2(b,c) show a twinning substructure in the deformed (engineering strain of 58%) AM SS 316 L specimen. Such deformation mode of twinning was previously observed by EBSD in AM SS 316 L and reported as the reason of the high strength and elongation^27,28,30^.

**Figure 3:**
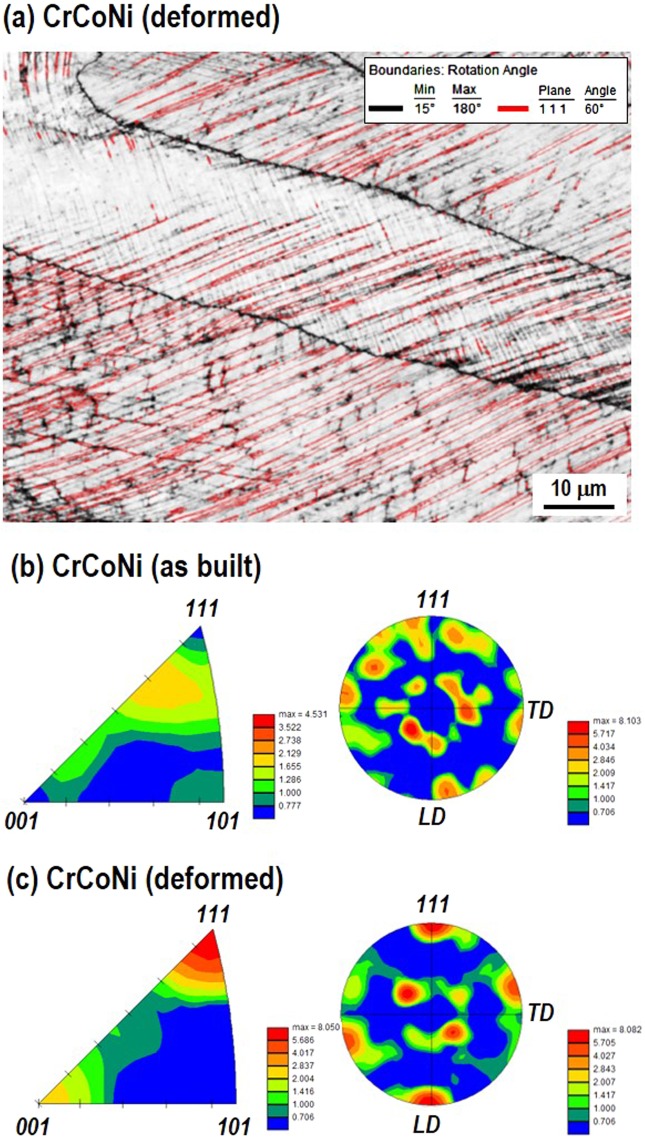
(**a**) EBSD grain boundary map imposed on the pattern quality image of Fig. 2f. Black is high angle grain boundaries and red indicates the misorientation angle of 60° rotation. Inverse pole figures along the longitudinal direction and pole figures of {111} in (**b**) as-built and (**c**) deformed AM CrCoNi specimens.

In the as-built AM CrCoNi, Fig. 2(d) shows also the columnar grain structure. It should be mentioned that the mean grain size is about 42 μm along x, which is relatively larger than typically found in cast-wrought annealed CrCoNi alloys (13–24 μm)^6–8^. Considering comparable tensile properties between cast-wrought and AM CrCoNi alloys, the dominant strengthening factor is highly relevant to the deformation twinning rather than the initial grain size in AM CrCoNi. Indeed, abundant deformation twins were observed in the deformed AM CrCoNi (Fig. 2(e,f), an enlarged part of Fig. 2(e)). Note that the twinning is mostly found in grains with their [111] poles parallel to the loading direction (LD, x) as shown in the IPF-x map of Fig. 2(f). Figure 3(a) shows an EBSD grain boundary map superimposed on the pattern quality image of Fig. 2(f) at the strain of 58%. It highlights that 85% of misorientation angles have 60° rotation about the [111] direction, which is known as Σ3 type twin boundaries^6,7,28^. Figure 3(b,c) compares the local grain texture between as-built and deformed AM CrCoNi specimens with the analysis area of 1142 × 856 μm^2^. Note that the specimens for texture analysis were the same to those of Fig. 2(d,e). Figure 3(b) shows a weak preferred orientation of the as-built specimen in the inverse pole figure along LD and (111) pole figure. Meanwhile, the deformed specimen, Fig. 3(c) shows most (111) plane normal mainly oriented to the LD (//loading direction) and it is over 8 times stronger than as-built state. The prominent < 111 > //LD fiber, e.g., brass and rotated copper texture components, has been known as the feature of deformation grains in twinning-induced plasticity (TWIP) steels^39^.

### Lattice strain evolution

**Figure 4:**
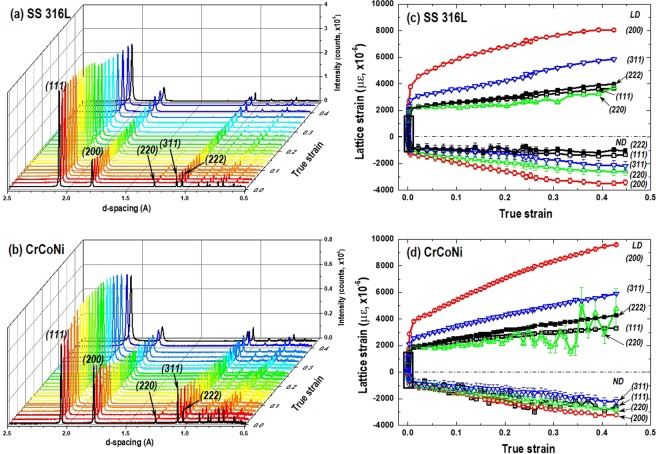
Diffraction peak patterns as a function of true strain collected at (**a**) AM SS 316 L and (**b**) AM CrCoNi specimens. Obtained by the axial detector where the scattering vector (Q) is paralleled to the longitudinal direction (LD). The evolution of lattice strains along the LD and normal direction (ND) measured from grain families of {111}, {200}, {220}, {311}, and {222} crystallographic planes during loading in (**c**) AM SS 316 L and (**d**) AM CrCoNi specimen.

Figure 4(a,b) shows peak patterns of neutron diffraction as a function of true strain under loading. Note that the shown peaks were diffracted from (*hkl*) grains with their plane normal parallel to the loading direction (Q//LD). All the measured diffraction peaks of raw data were shown in Fig. S3 (supplementary information). It shows the fcc structure and no phase transformation was observed as straining. Five grain families of (111), (200), (220), (311), and (222) were analyzed to determine each peak position, interplanar spacings (*d*_*hkl*_), and lattice strain (*ε*_*hkl*_). Figure 4(c,d) shows the lattice strain (*ε*_*hkl*_) evolution as a function of true strain. It shows an anisotropic intergranular lattice strain behavior, which is typical in fcc metals, for example, more harder grains in the orders of (200), (311), (111), and (220) along LD^40^. Linear fitting of the *ε*_*hkl*_ within the elastic region (0.1% of strain) can provide diffraction elastic constant (*E*_*hkl*_ = *σ*_*hkl*_ /*ε*_*hkl*_) and Possion’s ratio (*v*_*hkl*_ = −*ε*_*hkl*_^*ND*^/*ε*_*hkl*_^*LD*^) of each (*hkl*) plane (see Fig. S4). Table 1 shows larger (stiffer) *E*_*hkl*_ in AM CrCoNi than those of AM SS 316 L except *E*_200_. Note that the *v*_111_ for AM CrCoNi was inappropriate to determine due to the low intensity of the (111) peak followed by a scattered *ε*_111_ as shown in Fig. S4b. The *E*_*hkl*_ and *v*_*hkl*_ for the AM SS 316 L is comparable to the results of Krӧner elastic response modeling in fcc austenitic steel^40^. The bulk properties (*E*_*bulk*_ and *v*_*bulk*_) were obtained by *a*_*o*_ evolutions and others by *d*_*hkl*_. The *E*_*bulk*_ of 218 and 235 GPa for the AM SS 316 L and AM CrCoNi is slightly higher compared to the Young’s modulus of 193 and 229 GPa for the cast-wrought type SS 316 L and CrCoNi alloys in literature, respectively^4,28^.

### Stacking fault energy

**Figure 5:**
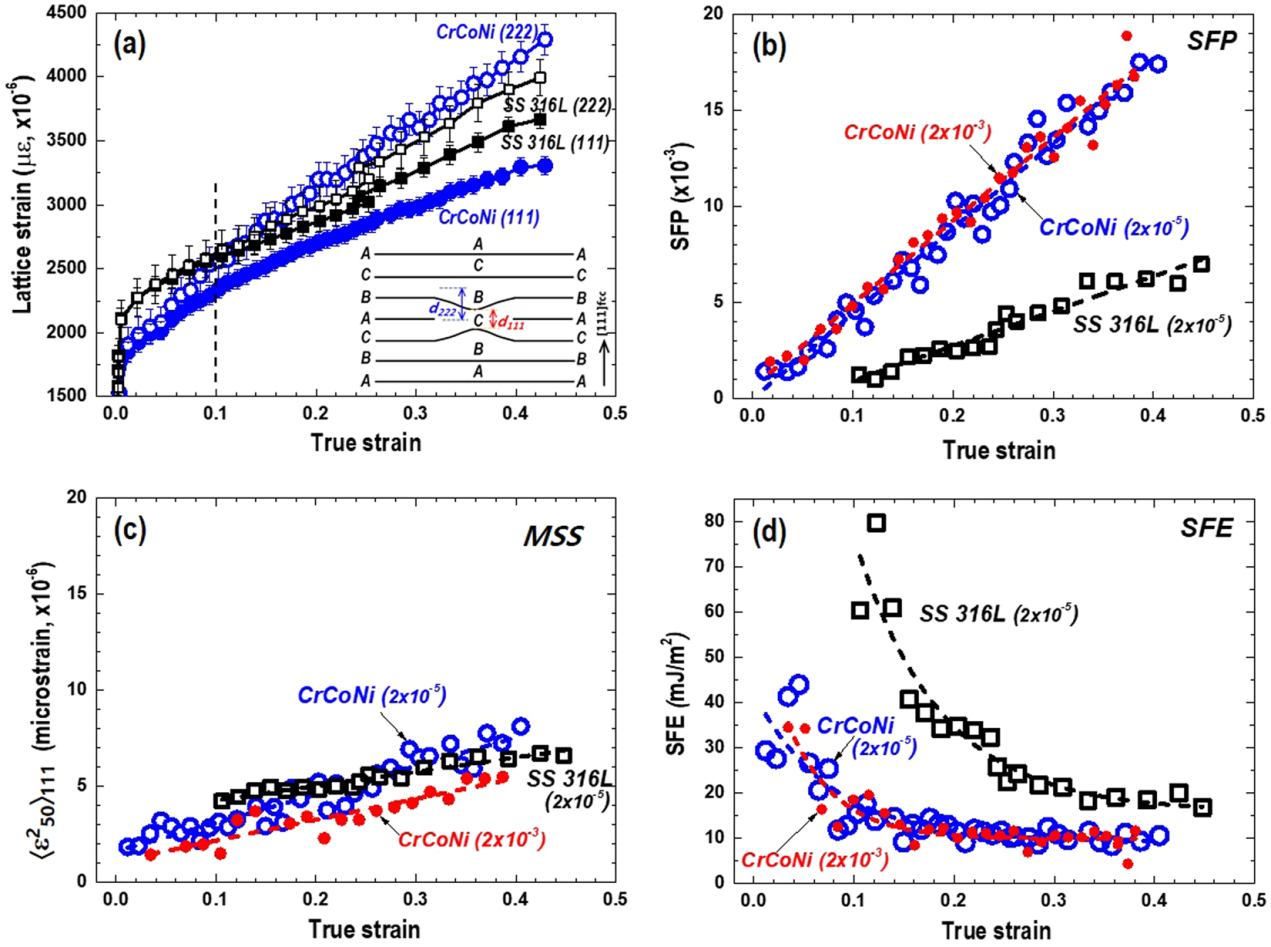
(**a**) Lattice strain evolutions of {111} and {222} crystallographic planes, (**b**) stacking fault probability (SFP, 10^−3^), (**c**) mean-square strain, (MSS, 〈*ε*^2^_50_〉_111_, 10^−6^), and (**d**) stacking fault energy (SFE, mJ/m^2^) as a function of true strain in AM SS 316 L and AM CrCoNi. Shown a schematic of lattice spacing changes due to an intrinsic stacking fault in inset of Fig. 5a. Included the low strain rate (LSR) of 2 × 10^−5^ s^−1^ and the high strain rate (HSR) of 2 × 10^−3^ s^−1^ cases.

As slip deformation occurs in fcc metals, (*a*/6)〈112〉 Shockley partial dislocations pass along the {111} plane and the behind region remains stacking faults^14^. The stacking faults have been known to affect diffraction peak shifts in opposite manner between (111) and (222) peaks in Eq. (2) (see equations in Methods)^41^. Plausibly, the *d*_222_ could be larger than *d*_111_ when a stacking fault exists as a schematic in the inset of Fig. 5(a). Indeed, Fig. 5(a) shows larger *ε*_222_ than *ε*_111_. The difference between *ε*_222_ and *ε*_111_ implies the creation of stacking faults as straining and more amounts occur in AM CrCoNi than AM SS 316 L. By correlating the *ε* difference to the SFP (*P*_*sf*_) in Eq. (3), Fig. 5(b) shows that the SFP increases significantly in AM CrCoNi and it is relatively three times higher compared to the AM SS 316 L. The physical meaning of 0.018 in SFP at the true strain of about 0.4 implies the existence of 18 stacking faults among 1,000 layers on average in 111-layers^42^. Note that the SFP below the true strain of 0.1 were unavailable to obtain in AM SS 316 L because of almost no difference between *ε*_222_ and *ε*_111_ in Fig. 5(a).

Figure 5(c) shows the MSS, 〈*ε*^2^_50_〉_111_, calculated by Eq. (8). The MSS of the two AM cases are similarly ranged in 4–7 μ*ε* (AM SS 316 L) and 3–8 μ*ε* (AM CrCoNi) within the corresponding true strain ranges. It suggests that relatively similar strains were induced by faulting and insignificant straining accumulation over the distance of 50 Å along the fcc [111] direction in both cases. Figure 5(d) shows the variations of the SFE as a function of true strain in AM SS 316 L and AM CrCoNi obtained by Eq. (1). The SFE of AM SS 316 L started 60–80 mJ/m^2^ at the true strain of about 0.1 and gradually decreases from 40 to 18 mJ/m^2^. Likewise, the SFE of AM CrCoNi analyzed initially about 40 mJ/m^2^ and decreases to ~10 mJ/m^2^. The averages of all SFEs at strains are 32.8 mJ/m^2^ for the AM SS 316 L and 15.1 mJ/m^2^ for the AM CrCoNi in Table 2. It is reasonable when compared to the previous results analyzed by TEM, x-ray, and neutron diffraction, for example, 12.9–42 mJ/m^2^ in SS 316 L^16–21^, 18–22 mJ/m^2^ in CrCoNi MEAs^9,11^, 27–32.5 mJ/m^2^ in CrCoNiFe HEA^11,12^, and 26.5–30 mJ/m^2^ in CrCoNiFeMn HEA^2,11^. Figure S5 (supplementary information) summarized the averaged SFE with its minimum and maximum in literature and current study.

**Table 2:** Stacking fault energies (SFE, mJ/m^2^) averaged at corresponding strain ranges in AM SS 316 L and AM CrCoNi alloys. The strain rates during tensile loading were marked in parenthesis.

SFE, γ (mJ/m^2^)				
Strain (*ε*)	SS316L (2 × 10^−5^)	Strain (*ε*)	CrCoNi (2 × 10^−5^)	CrCoNi (2 × 10^−3^)
0.10–0.45	32.8 ± 8	0.01–0.40	15.1 ± 4	13.3 ± 4
0.10–0.23	46.1 ± 10	0.01–0.12	23.9 ± 8	22.6 ± 8
0.23–0.45	20.8 ± 2	0.12–0.40	11.1 ± 2	10.4 ± 2

### Microstructure characterization by TEM

**Figure 6:**
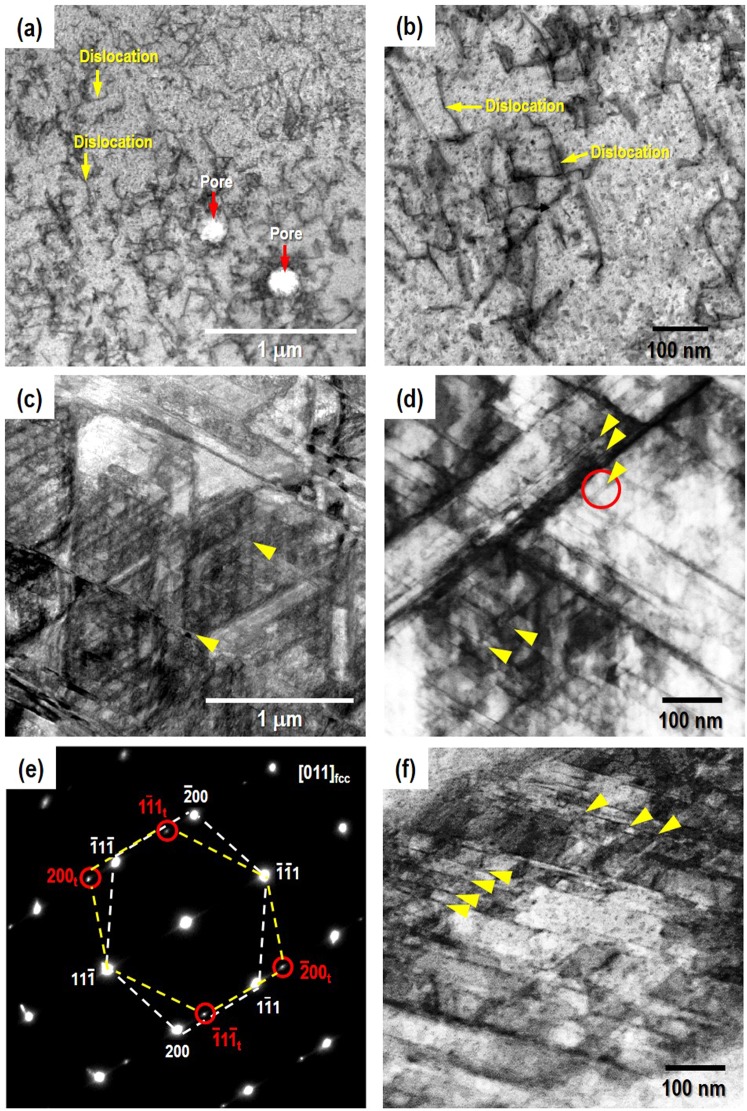
BF-STEM images along the 〈101〉 zone axis showing dislocations in as built (**a**,**b**) and multiple slip systems in deformed (engineering strain, *ε* = 58%) with LSR (2 × 10^−5^ s^−1^) (**c**,**d**) AM CrCoNi alloy. Marked twin spots in selected area diffraction (SAD) pattern (e) taken at the red circle in (**d**). Note that (**f**) is taken from deformed (*ε* = 47%) with HSR (2 × 10^−3^ s^−1^) AM CrCoNi alloy specimen.

Extensive TEM analyses have been reported in case of cast-wrought type stainless steels^16,18–21^ and CrCoNi alloys^5–9^. High resolution TEM images clearly show the nanoscale deformation twinning and strain-induced martensitic transformation in both alloys. Recently, a BF-STEM study of AM SS 316 L shows high density of dislocations and twins during plastic deformation^30^. It is consistent with the current observation in AM SS 316L (see Fig. S5 in supplementary information). For the AM CrCoNi specimens, Fig. 6 shows BF-STEM images of as-built (engineering strain of 0%, Fig. 6(a,b) and deformed (engineering strain of 58%, Fig. 6(c–e)) states. Figure 6(a,b) exhibits relatively low density of dislocations and a few pore defects in the initial stage of the AM CrCoNi specimen. The pore size is about 200 nm and mostly found along the interface of the scanning interlayers possibly due to entrapped vapor and/or lack of fusion during AM process^27^. Figure 6(c,d) shows multiple slip systems, which mean significant interactions between heavy dislocation densities and stacking faults including twin-twin interaction in the deformation substructure of the AM CrCoNi. Figure 6(e) is the selected area diffraction (SAD) pattern taken at the red circle in Fig. 6(d). It shows clearly the diffraction spots from the deformation twins and the fcc matrix with no other diffraction spots. Thus, it confirms that the deformation twinning is prevalent in the deformed AM CrCoNi specimen.

**Figure 7:**
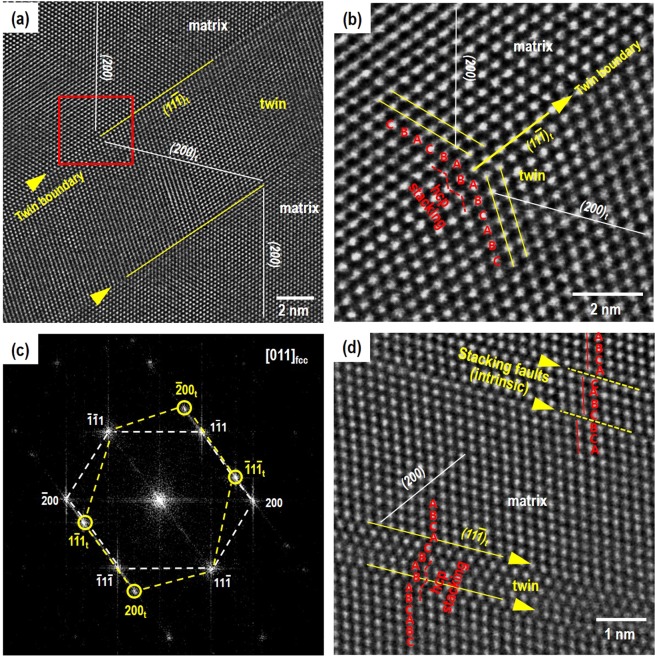
High-resolution HAADF-STEM images along the < 101 > zone axis of the AM CrCoNi specimen (**a**,**b**) deformed (*ε* = 58%) with LSR (2 × 10^−5^ s^−1^), (**c**) Fast Fourier transformation (FFT) of the TEM image of (**b**,**d**) deformed (*ε* = 47%) with HSR (2 × 10^−3^ s^−1^).

Figure 7 shows the HAADF-STEM images to elucidate detailed feature of the atomic stacking and subgrain boundaries in the deformed AM CrCoNi specimen. Figure 7(a) shows that stacking faults parallel to {111} planes create nano-twin substructures with the twin boundary distance of about 6.5 nm. Figure 7(b) is the enlarged image near the twin boundary marked a red square in Fig. 7(a). It shows the hcp stacking (BABAB) with the sharing of the same {111} habit plane (B stacking layer) as a coherent atomic matching nano-twin boundary. Similar hcp structure observed favorably within the nanotwins (so-called nanotwin-hcp lamella composite) has been reported in CrCoNi alloys^6,7^. Besides, Fig. 7(c) shows the fast Fourier transformation (FFT) reconstructed from the TEM image near the hcp stacking. There are clear spots by twinning and matrix, but does not detect the minor volume of the hcp phase.

## Discussion

### SFE variations as straining and critical twinning stress

**Figure 8:**
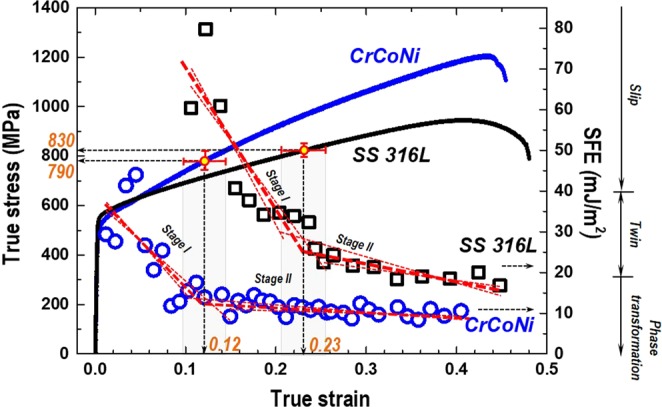
True stress-strain curves and stacking fault energies (SFE, mJ/m^2^) as a function of true strain. Significant variance of SFE is shown between stage I (initial) and stage II (stabilized). Linear fitting with adjacent points of 0.23 and 0.12 estimates the critical twinning stresses (*σ*_*tw*_) of AM SS 316 L and AM CrCoNi alloys, respectively. The strain rate is both 2 × 10^−5^ s^−1^. Typical dominant deformation mode in austenitic stainless steels was referred on side of the SFE.

Apparently the SFE decreases as a function of true strain in both the AM SS 316 L and AM CrCoNi alloy. Figure 8 shows that the SFE decreases from ~80 to 30 mJ/m^2^ (stage I) and stabilizes at about 20 mJ/m^2^ (stage II) from the strain of 0.23 in the AM SS 316 L. Similarly, the SFE varies from ~45 to 15 mJ/m^2^ (stage I) and fluctuates around 10 mJ/m^2^ (stage II) from 0.12 in the AM CrCoNi. The averaged SFEs of each stage as 46, 21 mJ/m^2^ (AM SS 316 L) and 24, 11 mJ/m^2^ (AM CrCoNi), respectively, as summarized in Table 2. Note that the critical strain values of the stage were determined at the significant variation of the slope (SFE/strain) by linear fitting. The reason of the transient SFE is attributed to the microstructure changes during deformation and its reflection on the faulting-embedded diffraction peak profiles. Several TEM studies have reported the evolution of the deformation substructure from planar dislocation slip to stacking faults, nanotwins, and hcp phase transformation in both austenitic steels^16,19^ and CrCoNi MEA alloys^6–9^. Besides, the current BF-STEM images clearly show the grain structure evolution of AM SS 316 L in Fig. S6 and AM CrCoNi MEA in Fig. 6. Consequently, a series of diffraction peaks from the variant microstructure as straining and the peak profile analyses enable us to provide faulting related parameters (SFP and MSS) and SFE variations at each strain. Thus, it is suggested that the SFE needs to be analyzed at various strain stages and averaged among the concerning strain range.

The critical twinning stress (*σ*_*tw*_) is described as an equivalent stress of importance to form sufficient stacking faults followed by the measureable deformation twins^8,37^. Several theoretical or phenomenological approaches have determined the *σ*_*tw*_ using TEM^8,9^, neutron diffraction^12^ or first-principle calculation based on energy barriers of stacking/twin faults^10,37^. It has been estimated for the CrCoNi alloy as 790 ± 100 MPa using TEM^9^, 890 MPa by first-principle calculation^10^, and 680–770 MPa by a numerical model by Steinmetz *et al*.^36^; *M*(*SFE*/3 *b*_*p*_ + 3*Gb*_*p*_/*L*_*o*_), where the *M* is the Taylor factor (3.13, mean value of Taylor factor map by EBSD, Fig. 2(d)) and assuming the *SFE* ranges 11–24 mJ/m^2^, *b*_*p*_ is the magnitude of the Burgers vector of partials (0.146 nm), *G* is the shear modulus (87 GPa), and *L*_*o*_ is the width of a twin embryo (200 nm)^9,12^. Besides, the Byun’ prediction^43^ reported the *σ*_*tw*_ of the SS 316 L of about 850 MPa with the SFE of 21 mJ/m^2^. Figure 8 shows significant variations of the SFEs at the strain of 0.12 and 0.23 between stage I and II. Supposedly it is relevant to the deformation substructure changes, the critical point of SFE is estimated to be located at 830 ± 25 MPa for the AM SS 316 L and 790 ± 40 MPa for AM CrCoNi. The stress ranges are comparable to the *σ*_*tw*_ of the cast-wrought type alloys above. The critical resolved shear stress for twinning (CRSS, *τ*_*tw*_ = *σ*_*tw*_*/M*) is suggested as about 260 MPa for the AM CrCoNi and 270 MPa for the AM SS 316 L with the *M* of 3.067^43,44^. Although similar *τ*_*tw*_, Fig. 8 shows that the AM CrCoNi (*ε* = 0.12) has a longer period of nano-twinning due to much earlier occurrence of the CRSS than the AM SS 316 L (*ε* = 0.23). Supposedly, higher shear modulus of the AM CrCoNi can also lead to higher work hardening rate and ultimate tensile strength as shown in Fig. 1(c,d). Note that reported shear modulus is 65.6 GPa for SS 316 L and 87 GPa CrCoNi alloy^9,43^.

### Effect of strain rates on SFE and deformation substructure

Let us discuss about the relationship between strain rates and SFE/microstructure in AM CrCoNi alloy. Firstly, it should be mentioned that higher strength properties were observed when tensile loaded with a higher strain rate. Note that an additional AM CrCoNi specimen was prepared with the identical sample dimension and tensile loaded at a relatively higher strain rate (HSR) of 2 × 10^−3^ s^−1^ compared to the lower strain rate (LSR) of 2 × 10^−5^ s^−1^. The stress-strain curve at the HSR results in the *σ*_*y*_ of 560 MPa, the *σ*_*UTS*_ of 850 MPa, and the *ε*_*f*_ of 47% (Fig. 1(c)), which are higher than LSR as summarized in Table 1. The observed stress relaxations of about 50 MPa (corresponding to about 10% of flow stress) is caused by interrupts with displacement holding for 600 s at each step of 0.5 mm in the plastic regime until fracture. It is necessary for the neutron diffraction *in situ* experiments to record diffraction peaks due to the long neutron counting time resolution (~a few minutes)^12^. Higher strengths readily achieved by HSR is likely relevant to the dominant stacking faults/twins in microstructure due to the excess of a critical stress^43^.

In this regard, twinning deformation structure of the HSR AM CrCoNi specimen need to be carefully examined. Figure 6(f) shows BF-STEM image of HSR strained of 47%. It shows significant twin-twin interactions in multiple slip systems. The number of twins are ~35% more intersected per unit length in HSR (Fig. 6(f)) than LSR (Fig. 6(d)) (by the grid line method in ref^8^.). Furthermore, the atomic scale HAADF-STEM image, Fig. 7(d), shows the twinning structure with the boundaries paralleled to {111} planes and the hcp stacking along the twin boundary, which is similar to the LSR AM CrCoNi, Fig. 7(a,b). Reported such hcp stacking acts as a favorable growth site for the hcp phase resulting in nanotwin-hcp lamella structure^6^. The twin spacing of ~1.5 nm was in HSR (Fig. 7(d)) is narrower than that of the LSR (~6.5 nm, 58% strained) (Fig. 7(a)). Considering the inverse relationship between the mean twin spacing (*t*) and the twin volume fraction (*f*_*tw*_) by the Fullman’s volumetric relationship; 1/*t* = (1/2*e*)·*f*_*tw*_/(1-*f*_*tw*_), where *e* is the average twin width, the narrower *t* implies the large amount of *f*_*tw*_ in HSR^36^. A TEM study reported decreased *t* and increased *f*_*tw*_ with similar *e* at large strains of CrCoNiFeMn HEA^8^. Thus, current atomic feature of the narrow twin spacing suggests that predominant stacking faults/twins can introduce new interfaces interacting with dislocations (so-called dynamic Hall-Petch effect) as straining and this phenomena could heavily involve the strengthening (higher flow stresses) in HSR AM CrCoNi (Fig. 1(c)). Besides, an intrinsic stacking fault (A/CABC/BC stacking) was found in Fig. 7(d), which was previously interpreted by using the fringe contrast method in dark-field TEM^9^.

The effect of strain rates on SFE can be discussed in AM CrCoNi alloy. In general it has been known that the SFE increases as increases temperature and/or concentration of alloying elements such as Ni, Al, Mn, C, N mainly due to the phase stability^18,45,46^. Meanwhile, the SFE decreases as increases grain size and alloying amounts of Si, Cr, Co in fcc structure metals and alloys^47,48^. In the current study, the mean SFE of the HSR (13.3 mJ/m^2^) is marginally lower than LSR (15.1 mJ/m^2^) in AM CrCoNi alloy as summarized in Table 2. It is possibly attributed to the large amounts of stacking faults/twins at HSR, which increase the SFP and in turn, drives the decrease of the SFE in Eq. (1). It is evident when examined higher SFP of ~10^−3^ with slightly lower MSS (~2 × 10^−6^) as a function of strain (Fig. 5(b,c)) in HSR AM CrCoNi alloy. Lastly, it seems to be insufficient of the adiabatic heating effect on the SFE caused by the current static strain rates (~10^−3^ s^−1^). Typically high strain rates may not provide enough time to dissipate heat from interior to surface of a tensile specimen and increase temperature and SFE. Benzing *et al*., reported a weak dependency of temperature and SFE on strain rates, for example, increase only 2.5 mJ/m^2^ by 17.2 °C increases when the strain rates increases from quasi-static (2 × 10^−4^ s^−1^) to low-dynamic (2 × 10^2^ s^−1^) spectrum in an austenitic high Mn TWIP steel^49^.

## Conclusions

1. Directed energy deposition process fabricated 17Cr-12Ni SS 316 L (additively manufactured SS 316 L) and 33Cr-35Co-34Ni medium entropy alloy (AM CrCoNi) specimens by the orthogonal scanning strategy using AM powders sized less than 150 μm using the process energy density of 71 J/mm^2^. Inverse pole figure (IPF) map shows that the initial columnar grain structure significantly changed to the deformation twinning in both specimens. Grain boundary quality imaging map shows that 85% of misorientation angles have 60° rotations about the [111] direction (Σ3 type twin boundaries) in the deformed AM CrCoNi specimen. The IPF and pole figures show most of (111) plane normals oriented to the loading direction resulting in 8 times stronger preferred orientation compared to the weak initial texture.
2. Tensile properties of the yield strength (*σ*_*y*_), ultimate tensile strength (*σ*_*UTS*_), and elongation (*ε*_*f*_) were 540 MPa, 660 MPa, 62% in the AM SS 316 L and 490 MPa, 800 MPa, 57% in the AM CrCoNi specimen, respectively. Those are enhanced or comparable to the properties of the typical cast-wrought type alloys. The work hardening rate (WHR, d*σ/dε*) of the AM CrCoNi remains mostly higher than that of the AM SS 316 L.
3. Neutron diffraction peaks show the single fcc structure with the initial lattice parameters (*a*_*o*_) of 0.3596 nm for the AM SS 316 L and 0.3567 nm for the AM CrCoNi alloy. In the elastic region, diffraction elastic constants (*E*_*hkl*_) and Possion’s ratios (*v*_*hkl*_) were obtained. In the plastic region, anisotropic behavior was observed among the intergranular lattice strains and the grains are typically harder in the orders of (200), (311), (111), and (220).
4. The stacking fault probability of the AM CrCoNi alloy clearly higher than that of the AM SS 316 L, whereas the mean-square strains is similar. It provides the apparent SFEs of 32.8 mJ/m^2^ for the AM SS 316 L and 15.1 mJ/m^2^ for the AM CrCoNi alloy. As straining, the SFE varies from 46 mJ/m^2^ (*ε* = 0.1–0.23) to 21 mJ/m^2^ (*ε* = 0.23–0.45) for the AM SS 316 L and 24 mJ/m^2^ (*ε* = 0.01–0.12) to 11 mJ/m^2^ (*ε* = 0.12–0.4) for the AM CrCoNi alloy. The critical twinning stresses are suggested as 830 ± 25 MPa for the AM SS 316 L and 790 ± 40 MPa for AM CrCoNi by analyzing stresses at a variant point of the SFE.
5. The reason of the transient SFE is relevant to the microstructure changes from dislocation slip to twinning deformation. Peak profile analyses of the faulting-embedded diffraction patterns provide the defect-related parameters at each strain. BF-STEM images exhibit low density of dislocations initially and as straining heavy dislocations with stacking faults and the twinning structure confirmed by the selected area diffraction (SAD) pattern. HAADF-STEM images elucidated the nano-twinning substructure paralleled to {111} planes and the atomic stacking of hexagonal close packing (hcp) structure in the deformed AM CrCoNi specimen.

## Methods

### Sample preparation by additive manufacturing

As-received commercial austenitic stainless steel powder (SS 316 L, 17Cr, 12Ni, 2.5Mo, 0.03 C, 0.75Si, 2.0Mn, 0.05 P and balance Fe, in wt.%) and pre-alloyed CrCoNi medium entropy alloy powder (CrCoNi HEA, 32.5Cr, 34.5Co, 33.5Ni, 0.031 O and 0.001 N in wt.%) were prepared as summarized in Table S1 (supplementary information). Plate type (54-mm long by 20-mm wide by 3-mm thick) of tensile specimens were additively manufactured by using the DED process (Fig. 1(a)). Denoted the longitudinal (LD, x), transverse (TD, y), and normal (ND//building, z) directions. The AM DED powder size, morphology, and chemical compositions were examined using a field emission scanning electron microscope (FE-SEM) equipped with energy dispersive spectrometry (EDS).

Using the AM powder with the particle size of 40–150 μm (Fig. 1(b)), the DED process was performed using a laser power of 380–400 W, a scanning speed of 14.1 mm/s, a powder feeding rate of 0.042 g/sec, a layer thickness of 250 μm, and a hatch spacing (laser beam spot size) of 400 μm under argon gas atmosphere with a pressure of 10 mbar and an oxygen of 0.2%, Table S2. The energy density (*E* = *P/dv*, where *P* is the power, *d* is the hatch pitch, *v* is the scanning speed) is about 71 J/mm^2^ and categorized to the middle-size DED process^29^. The scanning strategy was the orthogonal scan, which is firstly scanned with the vector along LD and secondly along TD starting from the same location among layers as shown in Fig. 1(a). Automatic feedback controlling system in a DED facility (INSSTEK MX-400) maintained the layer thickness and hatch width of the deposition by changing the laser power instantly.

### Microstructural characterization

Grain structure was examined on the parallel length part of the specimens along the TD (y) marked as an arrow in Fig. 1(a). Both AM SS 316 L and AM CrCoNi specimens were prepared before loading (as-built, engineering strain of 0%) and after fracture (deformed, engineering strain of ~58%). Each was cut from the grip region and 1 mm from the fractured edge at the mid-thickness, respectively, using electrical discharging machining (EDM). The surface of about 1 mm^3^ cube was mechanically grinded and polished down to the level of 0.02 μm colloidal silica suspension, then analyzed with the step sizes of 0.1–2 μm by the field emission scanning electron microscopy (FE-SEM, S-4300SE) equipped with EBSD system (e-Flash^HR^). Microstructures of as-built and deformed AM CrCoNi specimens were observed via TEM. TEM specimens were prepared by focused ion beam (FIB) milling method (FEI, Helios NanoLab 450) and analyzed using a field-emission TEM (FEI, Titan cubed G2 60–300) equipped with double spherical aberration correctors at an accelerating voltage of 200 kV. Bright-field scanning TEM (BF-STEM) and high-angle annular dark-field scanning TEM (HAADF-STEM) images were obtained for high resolution microscopy and atomic stacking examinations.

### *In situ* neutron diffraction

*In situ* neutron diffraction experiments were performed under the tensile deformation using the TAKUMI diffractometer equipped with a load frame in the Materials and Life Science Experimental Facility (MLF) of the Japan Proton Accelerator Research Complex (J-PARC)^50^. The prepared plate-type tensile specimen was installed and tensile loaded at a strain rate of 2 × 10^−5^ s^−1^ for both AM SS 316 L and AM CrCoNi. Two detectors located at ±90° to the incident beam and the tensile machine is oriented at a 45° angle to the incident beam (Fig. S1, Supplementary information). Time-of-flight (TOF) diffraction patterns were recorded by the two detector banks simultaneously with their scattering vectors (Q) parallel and transverse to the loading axis, respectively. Thus, LD and ND strain components of the specimen were measured from the corresponding lattice planes within the scattering volume of 125 mm^2^, which was defined by the 5 mm wide and 5 mm high incident slit, and the 5 mm wide receiving collimators. The tensile deformation was conducted by a step-load controlling manner with 600 s holding at each 30 MPa step in the elastic regime and by a continuous manner in the plastic regime until fracture.

The collected neutron diffraction data were analyzed using the Rietveld method as implemented by the Z-Rietveld program^51^. Rietveld method is defined as a structure analysis method for the whole peak adjustment between the characteristics of the experimental and calculated peak patterns associated with the crystallographic space group. The Rietveld structure refinement provides the interplanar spacings (*d*_*hkl*_) of the measured peak patterns diffracted from a set of (*hkl*) grains. Lattice strains (*ε*_*hkl*_) were calculated by (Δ*d*^*hkl*^/*d*_*o*_^*hkl*^), where Δ*d*^*hkl*^ = *d*^*hkl*^-*d*_*o*_^*hkl*^ and *d*_*o*_^*hkl*^ is the reference interplanar spacing that was initially measured before loading.

### Peak profile analysis and stacking fault energy (SFE)

The diffraction peak profile convolutes microstructural parameters such as stacking faults, dislocations, and twining^41,52^. Here, we adopt the peak profile analysis methodology for the determination of the SFE based on stacking fault probability (SFP) and mean-square strain (MSS). The well-established Reed and Schramm’s relationship correlates SFE to the ratio of MSS (〈*ε*^2^_50_〉_111_) to SFP (*P*_*sf*_)^53^.

1 $$SFE=\frac{6.6{a}_{o}}{\pi \sqrt{3}}{\left(\frac{2{C}_{44}}{{C}_{11}-{C}_{12}}\right)}^{-0.37}\frac{{\langle {\varepsilon }_{50}^{2}\rangle }_{111}}{{P}_{sf}}\left(\frac{{C}_{44}+{C}_{11}-{C}_{12}}{3}\right)$$

where *a*_*o*_ (nm) is the lattice parameter and *C*_*ij*_ is the elastic stiffness coefficient. The *a*_*o*_ of 0.3596 nm for AM SS 316 L and 0.3567 nm for AM CrCoNi were obtained by Rietveld analysis. The *C*_11_, *C*_12_, *C*_44_ were found as 216, 145, 129 GPa for AM SS 316 L and 249, 156, 142 GPa for AM CrCoNi, respectively^54,55^.

Firstly, Warren suggested the relationship between the SFP (*P*_*sf*_) and the diffraction peak shift based on the theory of defect scattering from stacking faults^41^. The stacking faults-induced peak shift (Δ*2θ*) causes the difference of lattice strains (*ε*_*hkl*_) between the successive orders, {111} and {222}, in face centered cubic (fcc) structure as below;

2 $$\Delta \left(2\theta \right)=\frac{90\sqrt{3}{P}_{sf}\,\tan \,\theta }{{\pi }^{2}}\frac{1}{{h}_{o}^{2}\left(u+b\right)}\sum _{b}\left(\pm \right){L}_{o}$$

where $\sum _{b}(\,\pm \,){L}_{o}/{h}_{o}^{2}(u+b)$ is a reflection quantity, +1/4 for (111) and −1/8 for (222) [38,43]. Thus, *ε* = −Δ(2θ)/2tanθ = Δ*d*/*d*_*o*_ drives the relationship between SFP and lattice strain difference between *ε*_111_ and *ε*_222_ as below;

3 $${P}_{sf}=\frac{32\pi }{3\sqrt{3}}\left[{\left(\frac{\Delta d}{{d}_{o}}\right)}_{222}-{\left(\frac{\Delta d}{{d}_{o}}\right)}_{111}\right]$$

Secondly, Warren and Averbach formulated the physically broadened line profile as Fourier transform coefficients (*A*), which is composed of size (*A*_*S*_) and distortion (*A*_*D*_) coefficients caused by mainly diffracting from coherent domains and microstrain within crystallites due to lattice imperfections, respectively^56^. Balzar *et al*. proposed the *A*_*S*_ and *A*_*D*_ contributions separately, and further developed a size-strain broadening model with a simple Voigt function as below^57,58^;

4 $${A}_{D}(L)\cong \exp (-2{\pi }^{2}{s}^{2}{L}^{2}\langle {\varepsilon }^{2}(L)\rangle )=\exp (-2L{\beta }_{LD}-\pi {L}^{2}{\beta }_{GD}^{2})$$

where *L* is the distance between diffraction planes in real space, *s* (1/*d* = 2sinθ/λ) is a variable in reciprocal space, *β* (=*β*(2θ)cosθ_o_/λ) is the integral breadth of the peak in unit of s (nm^−1^). *β*_*LD*_ and *β*_*GD*_ is Lorentian (*L*) and Gaussian (*G*) strain (distortion, *D*) integral breadths, respectively. Then, Eq. (4) correlates the MSS, 〈*ε*^2^*(L)*〉, to *β*_*LD*_ and *β*_*GD*_^57^;

5 $$\langle {\varepsilon }^{2}\left(L\right)\rangle =\left(\frac{{\beta }_{LD,hkl}}{{\pi }^{2}L}+\frac{{\beta }_{GD,hkl}^{2}}{2\pi }\right)\frac{1}{{s}_{hkl}^{2}}$$

The apparent integral breadths (*β*_*G*_ and *β*_*L*_) of the measured peak pattern are the convolution of the size (*β*_*GS*_ and *β*_*LS*_), strain (*β*_*GD*_ and *β*_*LD*_), and instrumental effects. This study followed double-Voigt size-strain analysis (so-called double-Voigt method) to de-convolute size and strain effects on the peak profiles^57–59^. This approach defines the strain broadening (*β*_*D*_) as diffracting angle dependent (*s* = d* = 1/*d, s*_*o*_ = 1/*d*_*o*_), while the size broadening (*β*_*S*_) is not^58^;

6 $${\beta }_{L}={\beta }_{LS}+{\beta }_{LD}\frac{{s}^{2}}{{s}_{o}^{2}},\,{\beta }_{G}^{2}={\beta }_{GS}^{2}+{\beta }_{GD}^{2}\frac{{s}^{2}}{{s}_{o}^{2}}$$

Noted detail consequences. The whole-peak fitting was performed by Thomson–Cox–Hasting (TCH) pseudo-Voigt function^60^ and obtained the Lorentian (*Η*) and Gaussian (*σ*^2^) peak width using Z-Rietveld refinement program^51^. Each peak position (*d*, nm) was converted from TOF (μs) using TOF = *C*_*o*_ + *C*_1_x*d* + *C*_2_x*d*^2^ with instrumental parameters (*C*). The apparent peak widths (*Η*, *σ*^2^) were subtracted by the instrumental broadening factors (*Η*_ins_ = *γ*_*o*_ + *γ*_1_*d* + *γ*_2_*d*^2^, *σ*_ins_^2^ = *σ*_*o*_^2^ + *σ*_1_^2^*d*^2^ + *σ*_2_^2^*d*^4^), which is consisting of instrumental width parameters (*γ* and *σ*). Then intrinsic integral breadths (*β*_*L*_ = *Hπ/*2, *β*_*G*_^2^ = *2πσ*^*2*^) of the specimen were obtained. It is necessary to convert the integral breadths from real space (*d*) to reciprocal space (d* = 1*/d*) for correction of the unit of *s* (nm^−1^) by following; *β*_L_* = *β*_*L*_*/C*_*1*_x*1/d*^2^, *β*_G_*^2^ = *β*_*G*_^2^*/C*_1_^2^x*1/d*^4^ (in case of *C*_1_ 〉〉 *C*_*o*_, *C*_2_). Thus, the double-Voigt method determines the unknown *β*_*LD*_, *β*_*GD*_^2^ (slopes) and *β*_*LS*_, *β*_*GS*_^2^ (intercepts) in Eq. (6) by plotting both *β*_*L*_ and *β*_*G*_^2^ as functions of *s* for (111) and (222) reflections, respectively;

7 $$\begin{array}{c}{\beta }_{LD}^{\ast }/{s}_{o}^{2}=[{({\beta }_{L}^{\ast })}_{222}-{({\beta }_{L}^{\ast })}_{111}]/(1/{d}_{222}^{2}-1/{d}_{111}^{2})\\ {\beta }_{GD}^{\ast \,2}/{s}_{o}^{2}=[{({\beta }_{G}^{\ast 2})}_{222}-{({\beta }_{G}^{\ast 2})}_{111}]/(1/{d}_{222}^{2}-1/{d}_{111}^{2})\end{array}$$

Thus, the MSS (〈*ε*^2^_50_〉_111_) defined as an inhomogeneous strain quantity due to faulting components averaged over the distance (L = 50 Å) along the fcc [111] direction can be determined by Eq. (5);

8 $${\langle {\varepsilon }_{50}^{2}\rangle }_{111}=({\beta }_{LD}^{\ast }/{s}_{o}^{2})/50{\pi }^{2}+({\beta }_{GD}^{\ast \,2}/{s}_{o}^{2})/2\pi $$

Finally, the SFE in Eq. (1) was determined by the SFP in Eq. (3) and the MSS in Eq. (8).

## Supplementary information

Supplementary Information.
