# Supplementary material for: Stacking Fault Energy Analyses of Additively Manufactured Stainless Steel 316L and CrCoNi Medium Entropy Alloy Using In Situ Neutron Diffraction
Source: Sci Rep. 2020 Jan 28;10:1350. doi: 10.1038/s41598-020-58273-3 (PMC6987211; doi:10.1038/s41598-020-58273-3)
Supplement: Supplementary file 1 — Supplementary Information. [file 41598_2020_58273_MOESM1_ESM.pdf]

Supplementary Information

Stacking fault energy analyses of additive manufactured stainless steel 316L and CrCoNi medium entropy alloy using in situ neutron diffraction

W. Woo [1], J.S. Jeong [2], D.-K. Kim [3], C.M. Lee [1], S.-H. Choi [4], J.-Y. Suh [5], S.Y. Lee [6], S. Harjo [7], T. Kawasaki [7]

[1] Neutron Science Center, Korea Atomic Energy Research Institute, Daejeon, 34057, Korea

[2] Materials Technology Development Team, Doosan heavy industries, Changwon, 44610, Korea

[3] School of Mechanical Engineering, University of Ulsan, Ulsan, 44610, Korea

[4] Department of Printed Electronics Engineering, Sunchon National University, Sunchon, 57922, Korea

[5] high Temperature Energy Materials Research Center, Korea Institute of Science and Technology, Seoul, 02792, Korea

[6] Department of Materials Science and Engineering, Chungnam National University, Daejeon 34134, Korea

[7] J-PARC Center, Japan Atomic Energy Agency, 2-4 Shirakata, Tokai, Naka, Ibaraki, 319-1195, Japan

Table S1. Nominal chemical compositions of the powder stainless steel 316L (SS 316L) and CrCoNi medium entropy alloy (CrCoNi) for the additive manufactured (AM) specimens.

Composition (wt. %)	*C*	*Si*	*Mn*	*P*	*S*	*Mo*	*Fe*	*Cr*	*Ni*	*Co*	*O*	*N*
Stainless steel (316L)	*0.03*	*0.75*	*2.00*	*0.05*	*0.03*	*2.50*	*65.65*	*17.00*	*12.00*	*-*	*-*	*-*
Medium entropy alloy (CrCoNi)	*-*	*-*	*-*	*-*	*-*	*-*	*-*	*32.5*	*33.5*	*34.5*	*0.031*	*0.001*

Table S2. Processing parameters of the direct energy deposition (DED) to fabricate the AM SS 316L and AM CrCoNi medium-entropy alloy specimens.

Processing parameters					
Laser power (W)	*Scanning speed (mm/s)*	*Powder feed rate (g/sec)*	*Layer thickness (mm)*	*Hatch pitch (mm)*	*Gas pressure (mbar)*
380-400	*14.1*	*0.042*	*0.25*	*0.40*	*10 mbar (Argon)*

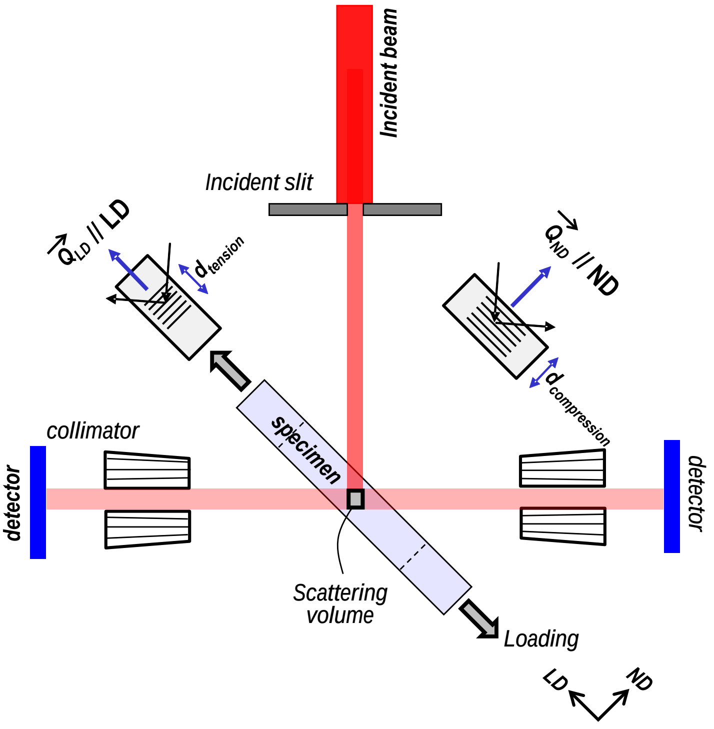

Figure S1. In situ neutron diffraction experimental set up. The tensile loading direction is 45° relative to the incident neutron beam direction with two detectors. Each detector measured lattice spacings (scattering vector, Q) parallel to the longitudinal direction (LD) and normal direction (ND) from the corresponding lattice planes within the scattering volume.

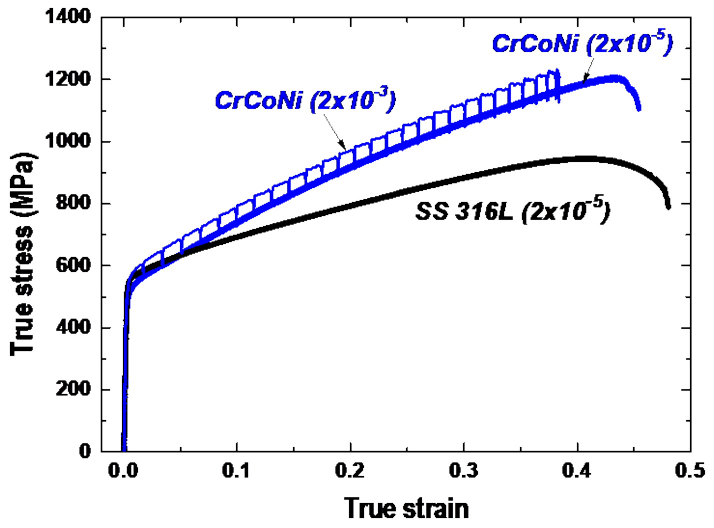

Figure S2. True stress-strain curves in tensile experiments with the strain rate of 2 x 10^{-5} s^{-1} for additive manufactured (AM) stainless steel 316L (SS 316L) and AM CrCoNi specimens and 2 x 10^{-3} s^{-1} for AM CrCoNi specimen.

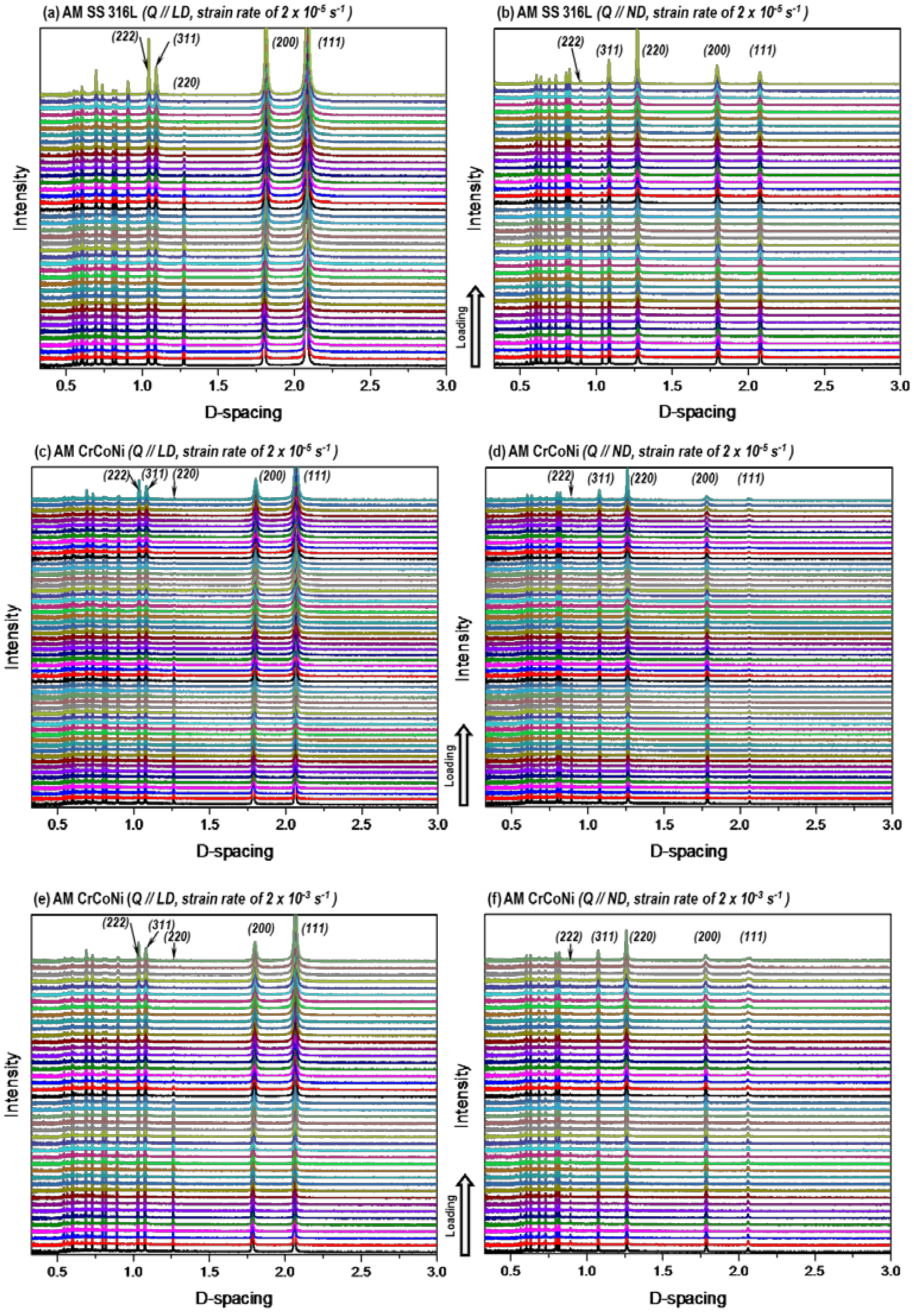

Figure S3. Neutron diffraction peak patterns under loading (raw data); (a) AM SS 316L (Q//LD), (b) AM SS 316L (Q//ND), (c) AM CrCoNi (Q//LD), (d) AM CrCoNi (Q//ND), (e) AM CrCoNi (Q//LD, strain rate of $2x10^{-3}$), (f) AM CrCoNi (Q//ND, strain rate of $2x10^{-3}$). Note that the diffraction patterns were obtained simultaneously by the two detectors where the scattering vector (Q) is paralleled to the longitudinal direction (Q//LD) and to the normal (Q//ND) direction. The strain rates of (a)-(d) is $2x10^{-5}$ and (e)-(f) is $2x10^{-3}$.

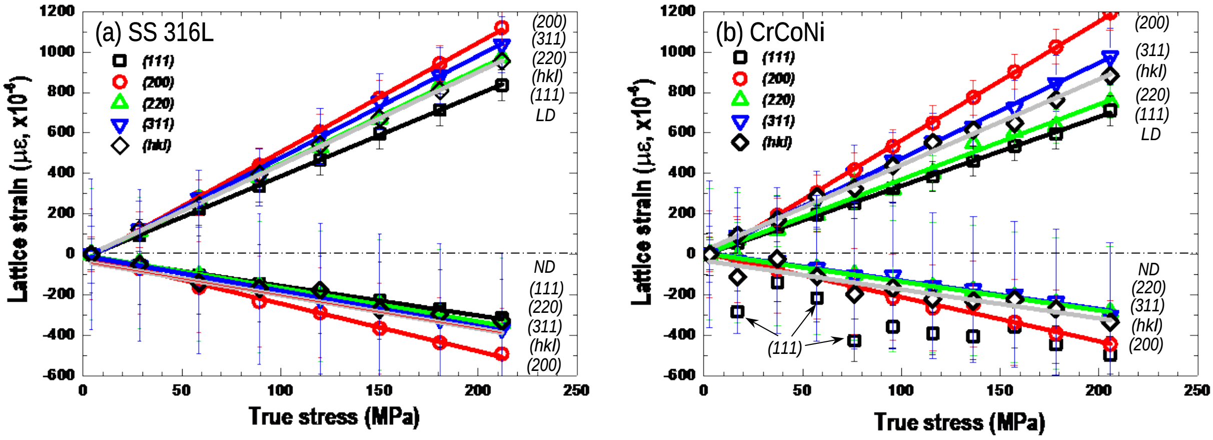

Figure S4. The evolution of lattice strains along the longitudinal direction (LD) and normal direction (ND) measured from grain families of {111}, {200}, {220}, {311}, and {222} crystallographic planes under loading (elastic region, 0.1% of strain); (a) AM SS 316L, (b) AM CrCoNi specimen.

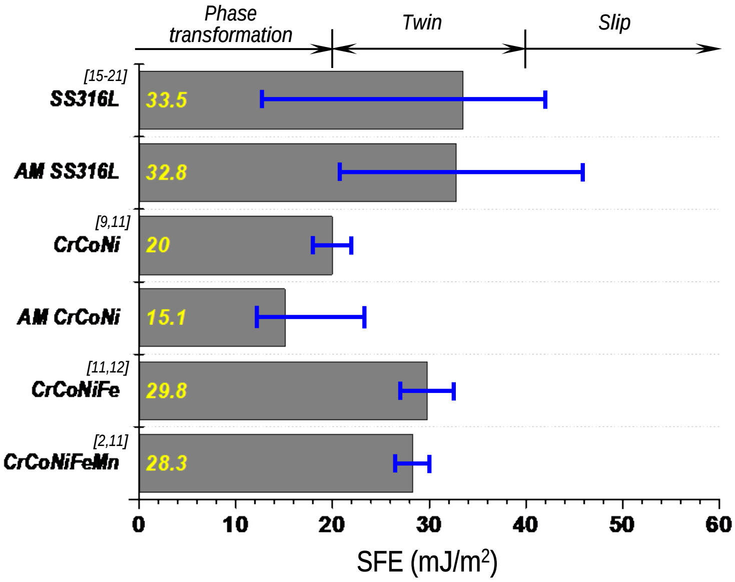

Figure S5. Summary of stacking fault energies (SFE, mJ/m^2) in cast-wrought type and additive manufactured (AM) SS 316L, CrCoNi, CrCoNiFe, and CrCoNiFeMn alloys. The presented SFEs were averages among reported results and the error bar was the minimum and maximum found in literature and current study. The typical dominant deformation mode for the austenitic stainless steel was presented on top for the reference.

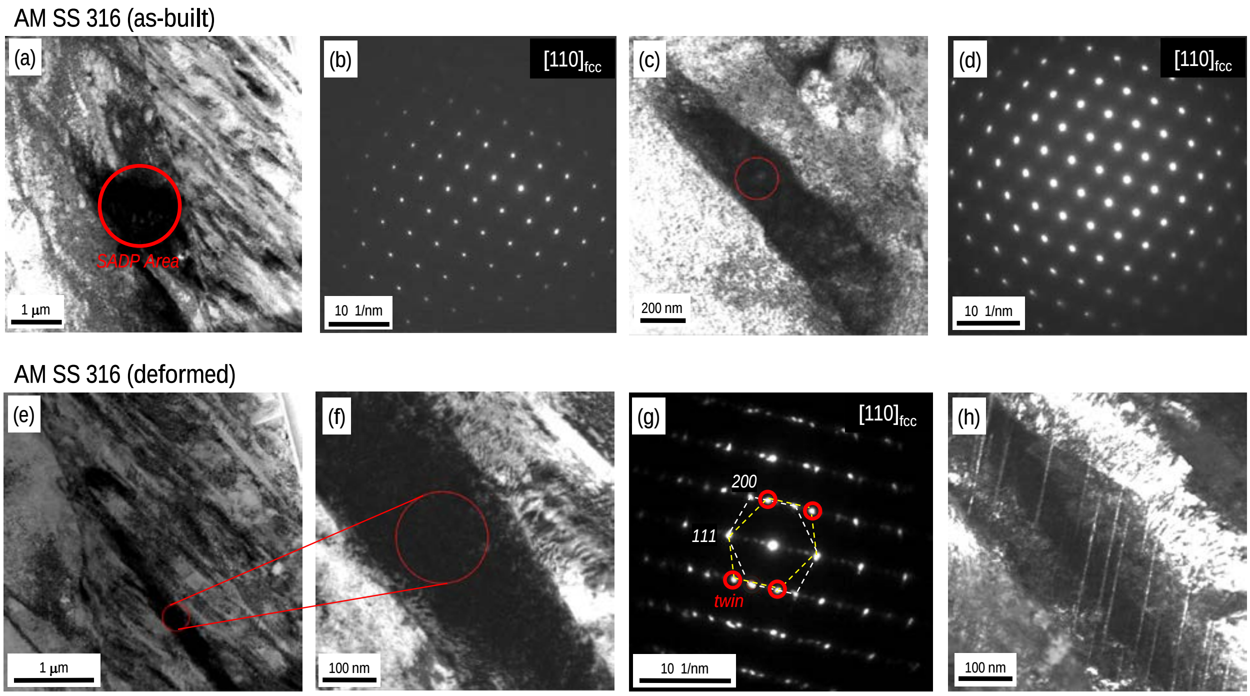

Figure S6. Bright-field transmission electron microscopy (BF-STEM) images of additive manufactured (AM) SS 316L specimen; (a)-(d) as-built and (e)-(h) deformed state, (a) and (c) BF-STEM, (b) and (d) selected area diffraction (SAD) pattern of each marked area, (e) and (f) BF-STEM, (g) SAD pattern of the marked area of (f), (h) twin deformation in BF-STEM.

Nominal chemical compositions of the powder stainless steel 316L (SS 316L) and CrCoNi medium entropy alloy (CrCoNi) for the additive manufactured (AM) specimens were summarized in Table S1. Processing parameters of the direct energy deposition (DED) to fabricate the AM SS 316L and AM CrCoNi medium-entropy alloy specimens were summarized in Table S2. Plate type (54-mm long by 20-mm wide by 3-mm thick) of tensile specimens were installed in neutron diffractometer diffraction measurements and tensile deformation were performed using a load frame as shown in Fig. S1.

Figure S2 shows the true stress-strain curve in AM SS 316L and AM CrCoNi specimens. It shows the yield strength (σ_y), ultimate tensile strength (σ_{UTS}), and elongation (ε_f) of 540 MPa, 945 MPa, 48% for the AM SS 316L and 485 MPa, 1210 MPa, 45% for the AM CrCoNi, respectively.

Figure S3 shows whole peak patterns of neutron diffraction under loading. Note that the shown peaks were diffracted from (hkl) grains with their plane normal parallel to the loading direction (Q//LD) and normal (Q//ND) direction of the specimen. Figure S4 shows that the linear fitting of the lattice strain obtained from the diffraction peak shifts within the elastic region (0.1% of strain). It can provide diffraction elastic constants (E_{hkl}) and Possion's ratio (v_{hkl}). Noticeably, the (111) peak intensity for the ND of AM CrCoNi was too low as shown in Fig. S3b. Thus, the v_{111} was unavailable to analyze appropriately.

Figure S5 shows a summary of the SFEs in literature and current study. The SFE of AM SS 316L is comparable to the ingot type SS 316L and the SFE of AM CrCoNi is slightly lower than that of the ingot type CrCoNi alloy. It can be due to the difference in the measurement method (TEM vs. neutron diffraction), processing method (cast-ingot vs. additive manufacturing), and/or analysis methodology (a few results at strain vs. a number of SFE data).

A recent BF-STEM analysis of AM SS 316L shows high density of dislocations and twins during plastic deformation[29]. Note that the utilized AM was the selective laser melting (SLM, a powder bed fusion process). Figure S6 is the results of direct energy deposition (DED) processed AM SS 316L and both results are comparable. The current study shows that the dislocation slip is dominant in as built state and deformation slip is profound in deformed DED AM SS 316L.
